# A deadly encounter: Alien invasive *Spodoptera frugiperda* in Africa and indigenous natural enemy, *Cotesia icipe* (Hymenoptera, Braconidae)

**DOI:** 10.1371/journal.pone.0253122

**Published:** 2021-07-16

**Authors:** Samira Abuelgasim Mohamed, Mark Wamalwa, Francis Obala, Henri E. Z. Tonnang, Tadele Tefera, Paul-Andre Calatayud, Sevgan Subramanian, Sunday Ekesi

**Affiliations:** 1 International Centre of Insect Physiology and Ecology (*icipe*), Nairobi, Kenya; 2 International Centre of Insect Physiology and Ecology (*icipe*), Addis Ababa, Ethiopia; University of Thessaly School of Agricultural Sciences, GREECE

## Abstract

The invasion and wide spread of *Spodoptera frugiperda* represent real impediments to food security and the livelihood of the millions of maize and sorghum farming communities in the sub-Saharan and Sahel regions of Africa. Current management efforts for the pest are focused on the use of synthetic pesticides, which are often economically unviable and are extremely hazardous to the environment. The use of biological control offers a more economically and environmentally safer alternative. In this study, the performance of the recently described parasitoid, *Cotesia icipe*, against the pest was elucidated. We assessed the host stage acceptability by and suitability for *C*. *icipe*, as well as its ovigenic status. Furthermore, the habitat suitability for the parasitoid in the present and future climatic conditions was established using Maximum Entropy (MaxEnt) algorithm and the Genetic Algorithm for Rule‐set Prediction (GARP). *Cotesia icipe* differentially accepted the immature stages of the pest. The female acceptance of 1^st^ and 2^nd^ instar larvae for oviposition was significantly higher with more than 60% parasitism. No oviposition on the egg, 5^th^ and 6^th^ larval instars, and pupal stages was observed. Percentage of cocoons formed, and the number of emerged wasps also varied among the larval stages. At initial parasitism, parasitoid progenies, time to cocoon formation and overall developmental time were significantly affected by the larval stage. Egg-load varied significantly with wasp age, with six-day-old wasps having the highest number of mature eggs. Ovigeny index of *C*. *icipe* was 0.53. Based on the models, there is collinearity in the ecological niche of the parasitoid and the pest under current and future climate scenarios. Eastern, Central and parts of coastal areas of western Africa are highly suitable for the establishment of the parasitoid. The geographic distribution of the parasitoid would remain similar under future climatic conditions. In light of the findings of this study, we discuss the prospects for augmentative and classical biological control of *S*. *frugiperda* with *C*. *icipe* in Africa.

## Introduction

Cereal crops, such as maize, *Zea mays* L. (Poaceae) and sorghum, *Sorghum bicolor* (L) (Poaceae) are the major staple food and source of income for millions of peoples in sub-Saharan and Sahel regions of Africa [1]. The production of these crops in Africa is hindered by several biotic and abiotic factors, chief among them is the infestation by insect pests [2–4]. The recent invasion of fall armyworm (FAW), *Spodoptera frugiperda* (J E Smith) (Lepidoptera: Noctuidae) has further compounded the problem. The aboriginal home range of *S*. *frugiperda* is the tropical and subtropical regions of the Western Hemisphere from Argentina to the United States of America [5, 6]. The pest was first detected in Africa in the rainforest zones of Nigeria in 2016 [7]. Subsequently, it has spread across sub-Saharan Africa [8], Egypt, Sudan, Mauritania, South and Southeast Asia, China [9] and recently in Australia [10]. The FAW is on the European and Mediterranean Plant Protection Organization (EPPO) A1 list and its spread northwards in Africa poses an eminent threat of invasion to Europe, the Middle East and beyond [11].

Fall armyworm is a polyphagous pest that attacks 353 wild and cultivated host plants belonging to 76 different families, with a preference to members of the family Poaceae, such as maize and sorghum [12–15]. Fall armyworm (FAW) represents a serious impediment to the production of these crops in Africa with far-reaching consequences on food security and livelihoods of millions of maize and sorghum farming households. A projection for yield losses of maize in 12 major maize producing countries in Africa indicates that crops worth over USD 13 billion per annum are at risk of FAW damage throughout sub-Saharan Africa, thereby threatening the livelihoods of millions of poor farmers in the continent [8, 16]. Alarmed by the spread and the magnitude of crop damage caused by *S*. *frugiperda*, national governments and farmers have resorted to the use of synthetic insecticides [10]. The frequent application of different classes of synthetic insecticides leads to significant increases in production costs, pest resistance development, increased health risks to the growers (in sub-Saharan Africa majority of whom are women) and consumers [16, 17].

Moreover, the use of synthetic insecticides will result in the disruption of integrated pest management (IPM) measures targeted at other pests in cereal cropping systems. The International Centre of Insect Physiology and Ecology (*icipe*) successfully managed the invasive stemborer *Chilo partellus* (Swinhoe) (Lepidoptera: Crambidae) through the release of the introduced parasitoid, *Cotesia flavipes* (Cameron) (Hymenoptera: Braconidae) [18–20] with estimated economic impacts of US$ 1.4 billion to the economies in Kenya, Mozambique and Zambia alone [21]. In this regard, there is a pressing need for an alternative eco-friendly IPM approach to tackle the FAW menace.

The habitat diversification approaches, ‘push–pull’ and maize–legume intercropping, are the most widely adopted in sub-Saharan Africa for management of maize pests. Recent evidence has highlighted the potential of both push–pull [22] and maize–legume intercropping [23, 24]. Diversified cropping systems are suitable for the conservation of natural enemies that can further contribute to the management of crop pests, such as fall armyworm [23]. Being an alien pest, it is imperative that classical biological control is an ideal option and could form the backbone of an IPM approach. Nevertheless, parasitoids that may have formed new association with FAW could contribute to its suppression and are worth considering. Indeed, the continent has a diverse fauna of natural enemies that are associated with other native *Spodoptera* spp [25–29] that can form new associations with FAW in Africa. In recent surveys conducted in eastern Africa, Sisay et al. reported larval parasitoids, such as *Charops ater* Szépligeti (Hymenoptera: Ichneumonidae), *Coccigydium luteum* (Saussure) (Hymenoptera: Braconidae), *Chelonus curvimaculatus* Cameron (Hymenoptera: Braconidae), *Palexorista zonata* (Curran) (Diptera: Tachinidae) [30] and egg parasitoids, such as *Telenomus remus* Nixon and *Trichogramma chilonis* Ishi [31]. Among these, *Cotesia icipe* Fernández-Triana & Fiaboe (Hymenoptera: Braconidae), was recovered in Ethiopia and Kenya and was the most dominant parasitoid in Ethiopia with parasitism levels of 33.8–45.3%. This study focused on assessing the ovigenic status of *C*. *icipe*, its acceptability of various instars of *S*. *frugiperda* for oviposition and the suitability of the immature stages of FAW for the development of *C*. *icipe*. Further, we modelled the habitat suitability for *C*. *icipe* globally and in Africa, under present and future climate change scenarios using maximum entropy algorithm.

## Materials and methods

### Host rearing

The colony of S. *frugiperda* was initiated with a cohort of 155 moths obtained from maize samples collected from Homa Bay County (Mbita and Ndhiwa, Kenya) and Siaya County (Rarieda and Alego Usonga, Kenya) and maintained in the laboratory at the Animal Rearing and Containment Unit (ARCU) at the International Centre of Insect Physiology and Ecology (*icipe*), Nairobi, Kenya. The rearing room was maintained at 25 ± 2°C, 60–70% RH and a photoperiod of 12L: 12D. Portable digital thermo-hygrometers were placed inside the rearing room to monitor temperature and relative humidity. The moths were held in Perspex cages (30x30x30cm) and provided with 20% honey solution with a moistened cotton wool ball placed in a Petri dish (8.6 cm in diameter).

The moths were provided with maize leaves obtained from a pesticide-free plant grown at *icipe*, for oviposition. Small pieces of leaves containing egg masses were removed from the rearing cage placed in a plastic jar (1.2 litres, 10 cm diameter, 16 cm height), and the hatching larvae were maintained until 3^rd^ instar. To minimise the cannibalism by 4^th^, 5^th^ and 6^th^; the 3^rd^ instar larvae were transferred to larger transparent plastic buckets (0.4 litres, 18 cm diameter, 21 cm height), where they were maintained till pupation. Pupae were periodically collected from the buckets (24–48 hrs) to avoid being cannibalised. The collected pupae were placed in Petri dishes and kept in an oviposition cage (30 × 30 × 30 cm) for moth emergence and oviposition. Periodically (3–4 months), *S*. *frugiperda* larvae were collected from the wild population and the emerging moths injected into the laboratory colony to maintain the genetic vigour.

### Parasitoid rearing

The initial cohort (14 wasps) of *C*. *icipe* colony was obtained from *Spodoptera littoralis* (Boisduval) (Lepidoptera: Noctuidae) parasitised larvae collected from *Amaranthus* plant in East and Central Kenya, specifically from Yatta (01.23044°S; 37.45789°E), Mwea (0.6309°S; 37.35117°E); Kitengela (1.6°S; 36.85°E) and Thika (1.00269°S; 37.07858°E). The parasitoid was reared on its natural host (*S*. *littoralis*) at *icipe* insectary for multiple generations. After that, a colony of the parasitoid was initiated using *S*. *frugiperda* in 2018, and maintained in the laboratory for five generation before conducting the bioassays. The parasitoid wasps were held in Perspex cages (30x30x30cm) kept in a rearing room under similar conditions as described above for host rearing. The wasps were fed with droplets of 20% honey solution placed on the inner topside of the rearing cage and water on moist cotton wool ball placed in Petri dish (8.6 cm in diameter). For colony maintenance, the wasps in the rearing cage were provided with early instar *S*. *frugiperda* on fresh maize leaves (cut into small pieces of about 10 cm). After 24 hrs, the exposed host larvae were removed from the cage and placed in rectangular plastic containers (20.5 cm length, 14.5 cm width, 8 cm height), containing fresh pieces of maize leaves for larval feeding. The larvae were maintained until parasitoid cocoon formation or pupation (in the case of unparasitised larvae). The formed cocoons were periodically removed from the leaves using a fine camel hairbrush and placed in a clean Perspex cage to allow new generations of adult parasitoids to emerge. The colony was maintained for three generations on *S*. *frugiperda* before the commencement of the bioassays.

### Host stage acceptability

The acceptability of different *S*. *frugiperda* immature stages; egg, L1, L2, L3, L4, L5, L6 (classified into instar based on age and colour as described by [32]) and pupa for oviposition by *C*. *icipe* was evaluated in the laboratory in a no-choice test. Six pairs (♀: ♂) of naive, 2–4-day-old wasps of the parasitoid were taken from the rearing cage and released in small cages (15 x 15 x 20 cm), one pair per cage, and provided honey and water as described above for parasitoid rearing. Ten larvae of each instar placed on pieces of maize leaves (~ 10 cm length) were offered to each pair of the wasp. After 8 hours, the exposed larvae were retrieved from each cage and larvae for each developmental stage were placed separately in a rectangular plastic container and provided with fresh maize leaves. On the following day of exposure, the larvae were dissected in phosphate buffer solution under a Leica Microsystems (Schweiz) AG stereomicroscope and the numbers of parasitised larvae, as well as the number of parasitoid egg(s) per larvae, were recorded for each larval instar. The experiment was replicated 10 times.

### Host stage suitability

The physiological suitability of *S*. *frugiperda* for the development of immature stages of *C*. *icipe* was assessed in the laboratory (set at the same conditions described above) under no-choice test. The experimental setup, including the host: parasitoid ratio and exposure duration is as described for the acceptability test. However, in this bioassay four wasps (1:1 ♀: ♂) and 20 of each larval instar (1^st^–6^th^) of *S*. *frugiperda* were used. After that, the exposed FAW larvae were removed from the experimental cages and each larval stage placed separately in rectangular plastic container and provided with fresh maize leaves where they were maintained till cocoon or pupal formation. Fresh maize leaves were periodically added to the feeding larvae in each container. The larvae were monitored daily and the formed cocoon(s) for each larval instar were removed using a soft camel hairbrush and placed in separate vials (25 x 150 mm). The number of the formed cocoon(s) and time to cocoon formation were recorded for each larval instar. The cocoons were further monitored, and adult parasitoid emergence recorded after every 24 hrs. The number of emerging wasps and their sex were recorded. The experiment was replicated 12 times for each larval instar.

### Egg load of *C*. *icipe*

The wasps used in this experiment were reared, as described above. Newly emerged naive unmated (0-d old) adult female wasps (n = 100) were collected from a laboratory-cultured *C*. *icipe* colony, placed in Perspex cages (30 x 30 x 30cm) and held under laboratory conditions (25 ± 2°C, 70–80 RH, 12:12 photoperiod). The wasps were fed with 20% honey solution as described above. On the day of dissection, 15 female wasps were randomly picked from the cage and transferred into a small Perspex cage (15 x 15 x 20 cm). The wasps (0, 3,6, and 9 days old) were dissected in a Petri dish (100mm x 15mm) with five drops of saline solution (0.9% NaCl solution) under a Leica Microsystems (Schweiz) AG stereomicroscope. Mature eggs from the ovaries and the lateral oviducts were counted and recorded separately for each wasp. The immature eggs that were mainly found in the distal portions of the ovarioles were not counted.

### Potential habitat suitability of *C*. *icipe*

The geocoded occurrence data of the parasitoid were derived from field surveys conducted in Kenya: Kilifi (3°2.2094S, 39°57.0234E - 3°10.34S, 39°58.759E); Kwale (4°19.837S, 39°20.603E); Taita Taveta (3°14.388S, 37°43.6E - 3°15.53S, 37°44.624E); Makueni (1°45.962S, 37°36.929E) and Machakos (1°9.171S, 37°25.91E - 1°45.714S, 37°28.842E). This data was augmented with georeferenced data from similar surveys carried out in Ethiopia and Tanzania [30, 31]. Environmental variables (such as temperature, rainfall), influence species distributions [33]. Current climate data (1960–1990) at 5-minutes high spatial resolution (approximately 9 km at the Equator) and future climate data for the year 2050 (2041–2060) were obtained from WorldClim database (http://www.worldclim.org/, Version 1.4) [34]. Bioclimatic variables (19) were extracted and used in combination with *C*. *icipe* occurrence records to assess current and future habitat suitability of the parasitoid [35, 36].

Maximum Entropy (MaxEnt) version 3.4.1 was chosen for species distribution modelling [37]. However, to better understand variation among bioclimatic factors, the MaxEnt and GARP via openModeller algorithms were used to develop models to estimate the potential geographic distribution of *C*. *icipe* under current and future climate scenarios [37, 38]. The occurrence data was partitioned into training data (75%) and test data (25%) whereby the training set was used to create the predictive model while the test set was used to assess model accuracy [39]. The modelling procedure assessed the importance of the environmental variables to *C*. *icipe* current and future habitat suitability through: (i) bootstrap analysis to determine the best model, with the number of replications equal to the number of samples [37]; (ii) jackknife analysis of the contribution of each variable to the model reliability when omitted; (iii) AUC analysis, where values of the receiver operator characteristic (ROC) plot were used to evaluate model performance whereby the model with the highest AUC value was considered the best performer; (iv) applying the “10 percentile training presence logistic threshold” to create suitability maps for species distribution. For visualisation and further analysis, the results of the MaxEnt models predicting the presence of *C*. *icipe* were imported into DIVA-GIS version 7.5 to prepare species distribution maps using an alternative classification method [40].

### Statistical data analysis

Percentage data on parasitism, female progeny (sex ratio), cocoons formed, and wasp emergence were arcsine square root transformed to stabilise the variance before subjecting to an analysis of variance (one-way ANOVA) to test the effect of the larval stage on these response variables. Time to cocoon formation was analysed using one-way ANOVA, while the total developmental time was analysed using two-way ANOVA, with larval stage and wasp sex as factors. Data on egg load were log-transformed (log_e_) before ANOVA. The Ovigeny Index (OI) was calculated as the ratio of the initial egg load to the potential lifetime egg complements as defined by Jervis [41]. Means were separated using Tukey’s HSD test at α = 0.05. The analyses were implemented in R version 3.3.3 [42].

## Results

### Host stage acceptability

**Fig 1:**
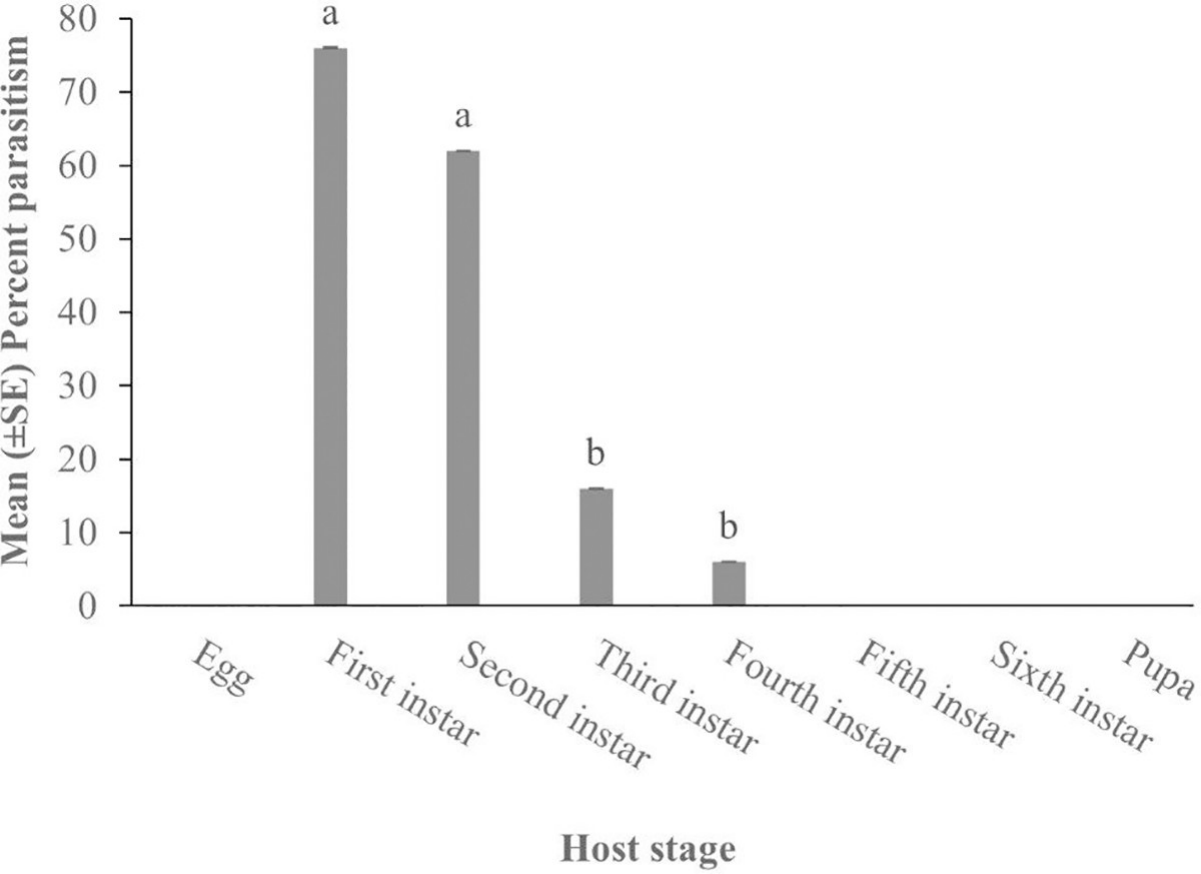
Acceptability of various immature stages of *Spodoptera frugiperda* for oviposition by *Cotesia icipe*.

*Cotesia icipe* differentially accepted the immature stages of *S*. *frugiperda* for oviposition (*F*_3,16_ = 19.98, *P* < 0.0001). The females accepted more 1^st^ and 2^nd^ instars with parasitism levels of more than 60%, followed by the 3^rd^ instar, while the 4^th^ instar was the least accepted for oviposition as shown in supplementary file S1 File. On the other hand, the egg, 5^th^ and 6^th^ instars and pupal stage were not accepted for oviposition by *C*. *icipe* females (Fig 1).

### Host stage suitability

Among the larval stages accepted for oviposition, the percent cocoons formed varied (*F*_3,38_ = 29.27, *P* < 0.0001) with the age of the instar, with 1^st^ and 2^nd^ yielding the highest. In contrast, the 4^th^ instar had the lowest number of parasitoid cocoons (Table 1). Among the cocoons formed, the mean number of parasitoids that emerged were significantly different across the four instars (*F*_3,34_ = 34.17, p < 0.0001). Similarly, the per cent female progeny significantly varied among the four instars (*F*_3,34_ = 12.37, *P* < 0.0001) with 1^st^, 2^nd^ and 3^rd^ instars producing more female progeny than instar 4 (Table 1). The host suitability data is available as supporting information in S2–S4 Files.

**Table 1:** Host stage suitability of *Spodoptera frugiperda* for immature development of *Cotesia icipe*.

Host stage	Parasitoid cocoon formed (%)	Number of emerged wasps	Female progeny (%)
First larval instar	69.1 ± 5.3a	12.0 ± 0.94a	64.2 ± 4.39a
Second larval instar	58.5 ± 4.2ab	10.6 ± 0.74a	68.3 ± 4.06a
Third larval instar	43.0 ± 7.23b	5.5 ± 0.97b	56.8 ± 6.84a
Fourth larval instar	5.63 ± 2.74c	1.3 ± 0.48c	12.5 ± 12.5b

### Developmental time

Larval instar at the initial parasitism by *C*. *icipe* had a significant effect on the time taken to cocoon formation (*F*_3, 330_ = 19.57, P <0.001), whereby *C*. *icipe* took a significantly longer time to cocoon formation on 4^th^ instar. No significant interaction between larval stage at initial parasitism and sex of emerging wasp (*F*_3,340_ = 0.47, P = 0.706) was seen. On the other hand, the main factor of larval instar at parasitism had a significant effect on wasp developmental time (*F*_3,340_ = 4.16, *P* = 0.006), with parasitism at 4^th^ larval instar resulting in the longest development time of 14.8 days as compared to 13 days for the other three larval stages as shown in supplementary file S5 File. Similarly, developmental time varied with wasp sex (*F*_1, 340_ = 8.51, *P* = 0.004), whereby females took a slightly longer time to emerge as shown in supporting information S6 File.

Comparing the developmental duration of different sexes reared on the same larval instar of FAW, there was no significant difference except for 4^th^ instar, whereby females took a longer time (Table 2).

**Table 2:** Mean (±SE) preimaginal developmental duration (days) of *Cotesia icipe* reared on various larval instars of *Spodoptera frugiperda*.

Host stage	Time to cocoon formation	Cocoon to adult emergence		
		Overall	Sex	
			Male	Female
First larval instar	7.91 ± 1.0^a^	12.82 ± 1.2^a^	12.37 ± 1.2^aA^	13.07 ± 1.2^aB^
Second larval instar	8.62 ± 1.2^ab^	12.93 ±1.3^a^	12.49 ± 1.2^aA^	13.21 ± 1.2^aB^
Third larval instar	8.36 ± 0.5^b^	12.62 ± 0.8^a^	12.52 ± 0.8^aA^	12.67 ±0.9^aA^
Fourth larval instar	10.11 ± 0.3^c^	14.13 ± 2.5^b^	12.25 ±2.1^aA^	16.00 ± 0.8^bB^

^1^Means in the same column followed by the same superscript lower-case letter are not significantly different (α = 0.05).

^2^Means in the same row followed by the same superscript upper-case letter are not significantly different (α = 0.05, Student t-test).

### Potential fecundity and ovigeny index

**Fig 2:**
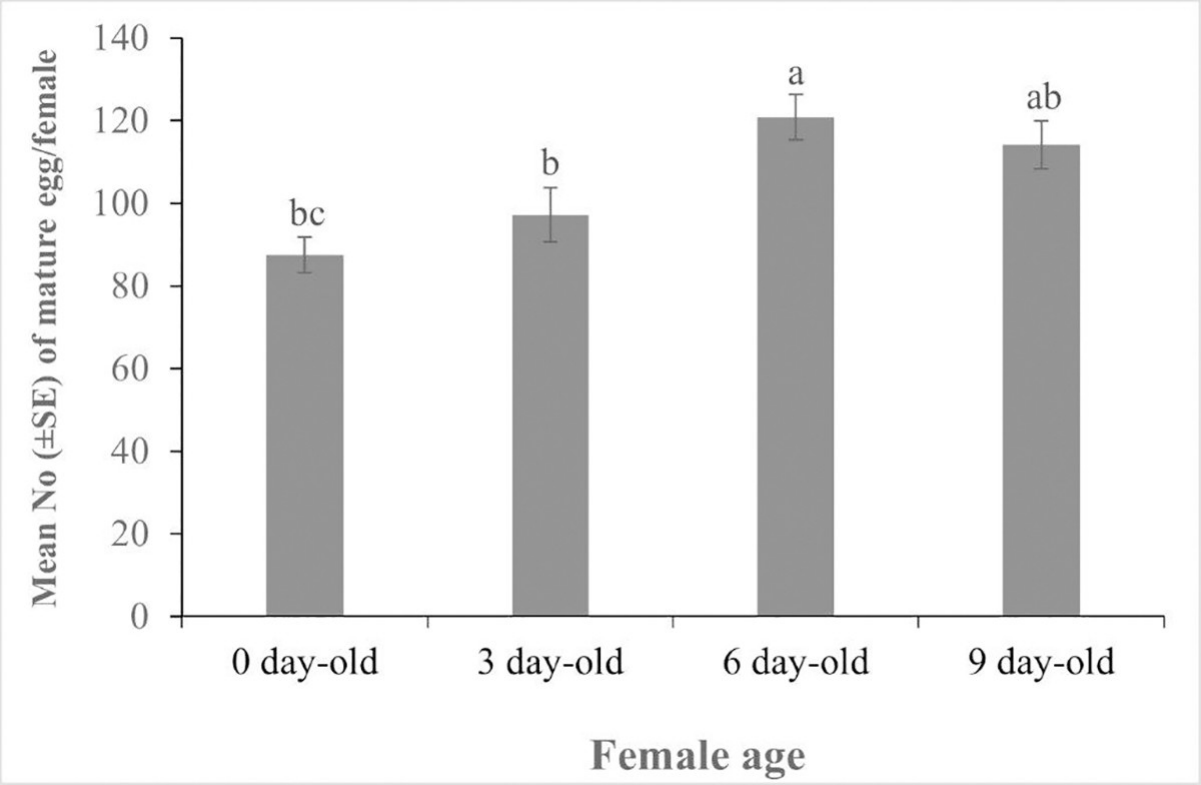
Egg load of naive *Cotesia icipe* female at different ages.

While newly emerged wasps (day 0) had a substantial number of eggs, egg-load varied remarkably with the wasp’s age (*F*_3,56_ = 6.92, *P* = 0.001). Six-day-old wasps had the highest complement of mature eggs. Although egg load declined for the nine-day-old wasps, it was still higher than that for zero and three-day-old wasps (Fig 2). Ovigeny index (OI) for *C*. *icipe*, computed as the ratio of the initial egg load to the potential lifetime egg complements, was 0.53. The potential fecundity and ovigeny data is available as S7 File.

### Species distribution modelling

MaxEnt version 3.4.1 [35] was chosen for species distribution modelling (SDM) because it can handle presence-only data and small sample sizes [37]. Bioclimatic variables with a high Pearson correlation coefficient (r ≥ 0.85) were eliminated from further models to reduce potential over-parameterisation [33, 43]. Subsequently, the MaxEnt model (AUC = 0.997 ± 0.001) identified four environmental variables as being significantly associated with *C*. *icipe* habitat suitability. Maximum temperature of the warmest month (Bio5) contributed most to the model, followed by isothermality (Bio3), precipitation of driest month (Bio14) and precipitation of wettest month (Bio13), respectively (Table 3). These four factors cumulatively accounted for 91.3% contribution to the model.

**Table 3:** Environmental variables used in the study and their percentage contribution to the *Cotesia icipe* Maxent model.

Variable	Percent contribution	Permutation importance
Maximum temperature of warmest month (Bio5)	50.4	10.0
Isothermality (Bio3)	15.0	18.0
Precipitation of driest month (Bio14)	13.9	71.2
Precipitation of wettest month (Bio13)	12.0	0.1
Mean Temperature of Coldest Quarter (Bio11)	3.4	0.0
Mean Temperature of Driest Quarter (Bio9)	3.1	0.4
Precipitation of Warmest Quarter (Bio18)	0.9	0.0
Precipitation of Coldest Quarter (Bio19)	0.6	0.2
Temperature Annual Range (Bio7)	0.6	0.0
Mean Diurnal Range in Temperature (Bio2)	0.1	0.1

### Potential distribution of *C*. *icipe*

The *C*. *icipe* parasitoid models (GARP and MaxEnt) predicted high habitat suitability in Kenya (Coastal, Eastern, Rift Valley, Mt. Kenya and Lake regions) and Ethiopia (Jimma, Borena, East Shewa, Hararghe and Tigray) where the parasitoid already prevails (Table 4).

**Table 4:** Future climatic suitability and its predicted accuracy for *Cotesia icipe*.

Location	Climate suitability	Prediction accuracy	*C*. *icipe* establishment
Kilifi, Malindi, Kenya	Yes	Yes	N/A
Kilifi, Malindi, Kenya	Yes	Yes	N/A
Kilifi, Magarini, Kenya	Yes	Yes	N/A
Kwale, Matuga, Kenya	Yes	Yes	Sisay et al., 2018
Taita Taveta, Chala, Kenya	Yes	Yes	Sisay et al., 2018
Taita Taveta, Chala/Njukini, Kenya	Yes	No	Sisay et al., 2018
Makueni, Kenya	Yes	Yes	N/A
Machakos,Yatta, Kenya	Yes	Yes	N/A
Machakos,Yatta, Kenya	Yes	Yes	N/A
Machakos,Yatta, Kenya	Yes	Yes	N/A
Hawassa, Ethiopia	Yes	Yes	Sisay et al., 2018
Jimma, Ethiopia	Yes	Yes	Sisay et al., 2018
Awash-Melkasa, Ethiopia	Yes	Yes	Sisay et al., 2018

**Fig 3:**
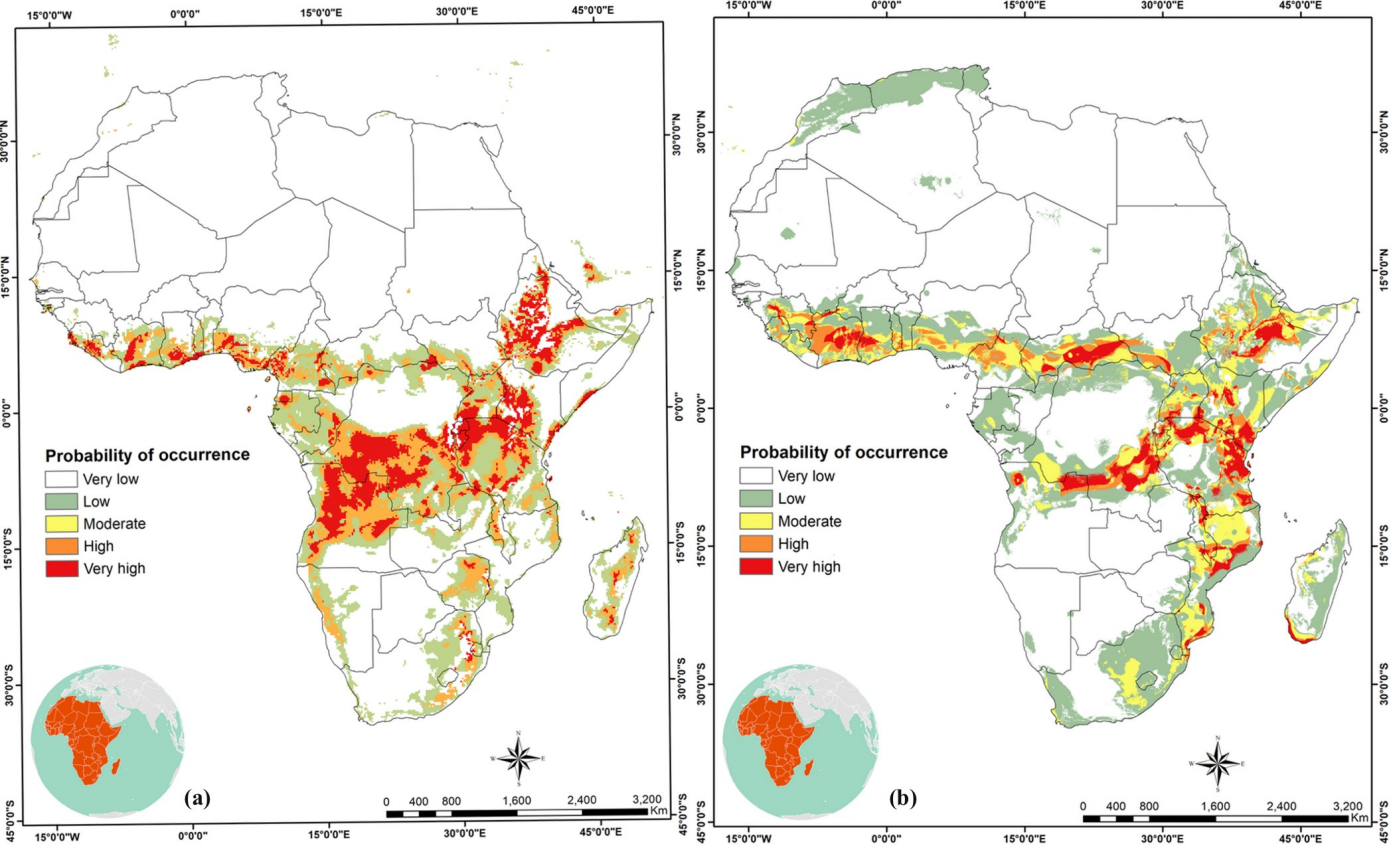
Current habitat suitability of *Cotesia icipe* in Africa. Potential distribution of *C*. *icipe* in Africa under current climatic conditions: (a) MaxEnt model current species distribution projections in Africa; (b) GARP model current species distribution projections in Africa. “The figure was generated using the MaxEnt 3.4.1 software (https://biodiversityinformatics.amnh.org/open_source/maxent/) and GARP (http://openmodeller.sourceforge.net/)” [38].

Under the current climate, potential areas of high habitat suitability for *C*. *icipe* increased towards eastern Africa (Eritrea, Tanzania, Southern Uganda, Rwanda and Burundi) and Central Africa (D.R. Congo, Equatorial Guinea and Cameroon). Western Africa regions of high suitability are restricted to the coastal belts of Ghana, Nigeria, Togo, Sierra Leone, Liberia and Ivory Coast. For southern Africa, high suitability areas are in Angola, while low to moderate suitability was predicted in Zambia, Zimbabwe, Madagascar and South Africa (Fig 3A and 3B).

**Fig 4:**
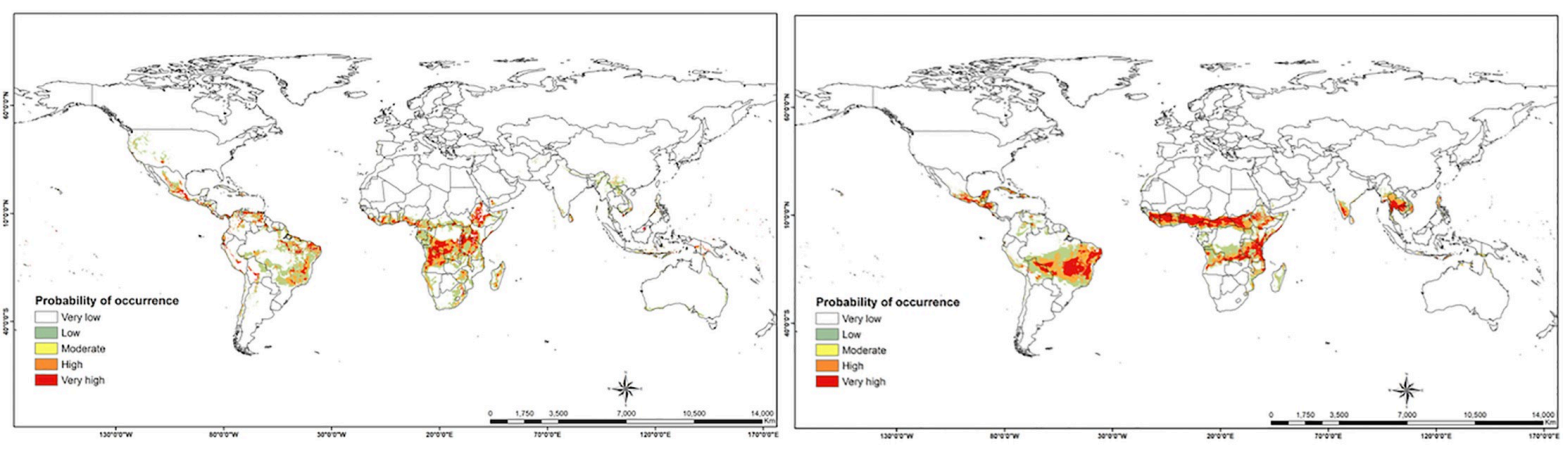
Current global habitat suitability of *Cotesia icipe*. Global potential distribution of *C*. *icipe* under current climatic conditions: (a) MaxEnt model current global species distribution projections; (b) GARP model current global species distribution projections. “The figure was generated using the MaxEnt 3.4.1 software (https://biodiversityinformatics.amnh.org/open_source/maxent/) and GARP (http://openmodeller.sourceforge.net/)” [38].

Globally, several countries in South and Southeast Asia and Australia are low to moderately suitable for the establishment of the parasitoid. High suitability for the establishment of *C*. *icipe* is also predicted in southern Mexico, Central America and several countries in southern America (Fig 4A and 4B).

**Fig 5:**
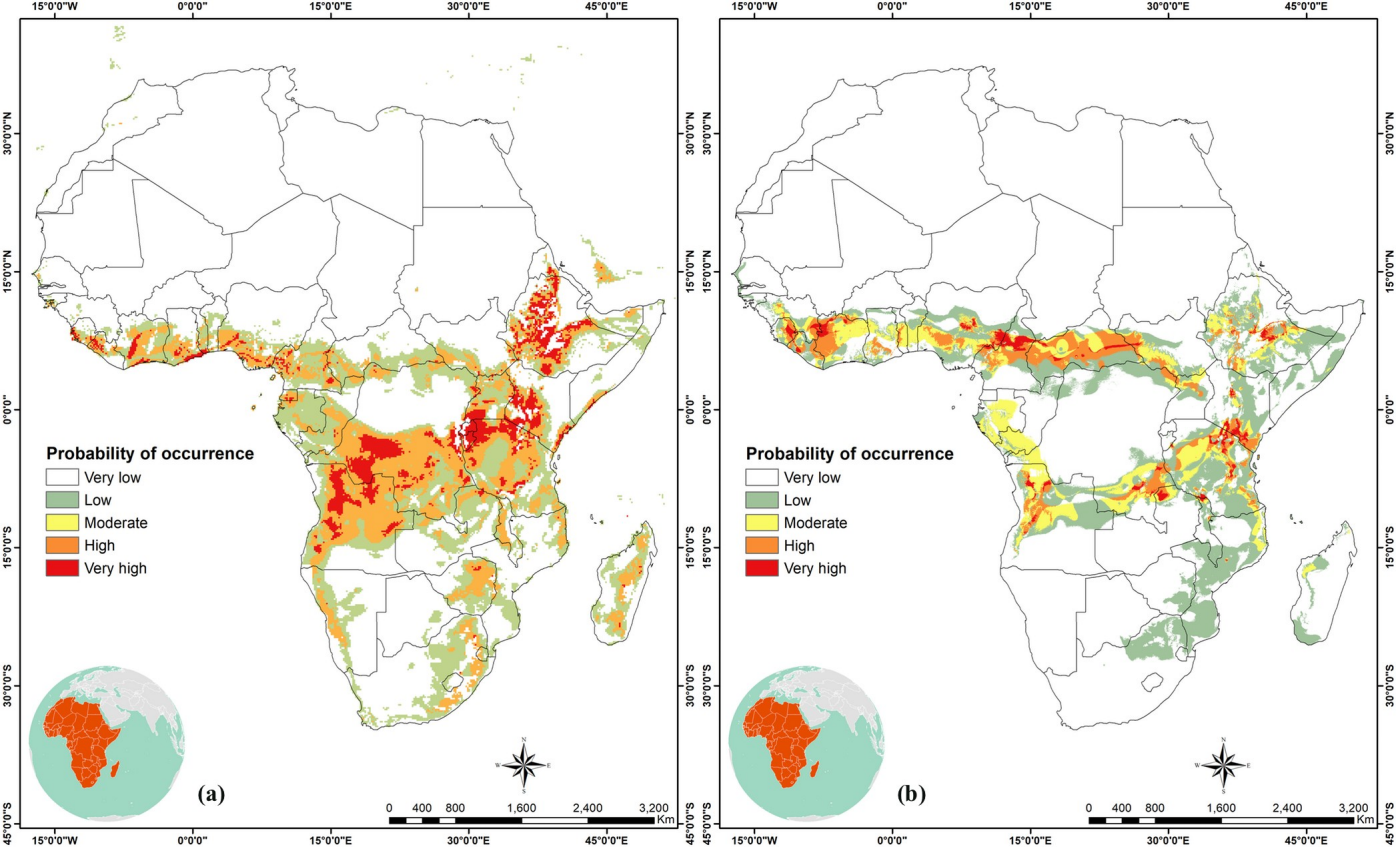
Future habitat suitability of *Cotesia icipe* in Africa. Predicted future potential distribution of *C*. *icipe* in Africa under climate change scenario: (a) MaxEnt model future species distribution projections in Africa; (b) GARP model future species distribution projections in Africa. The figure was generated using the MaxEnt 3.4.1 software (https://biodiversityinformatics.amnh.org/open_source/maxent/) and GARP (http://openmodeller.sourceforge.net/) [38].

**Fig 6:**
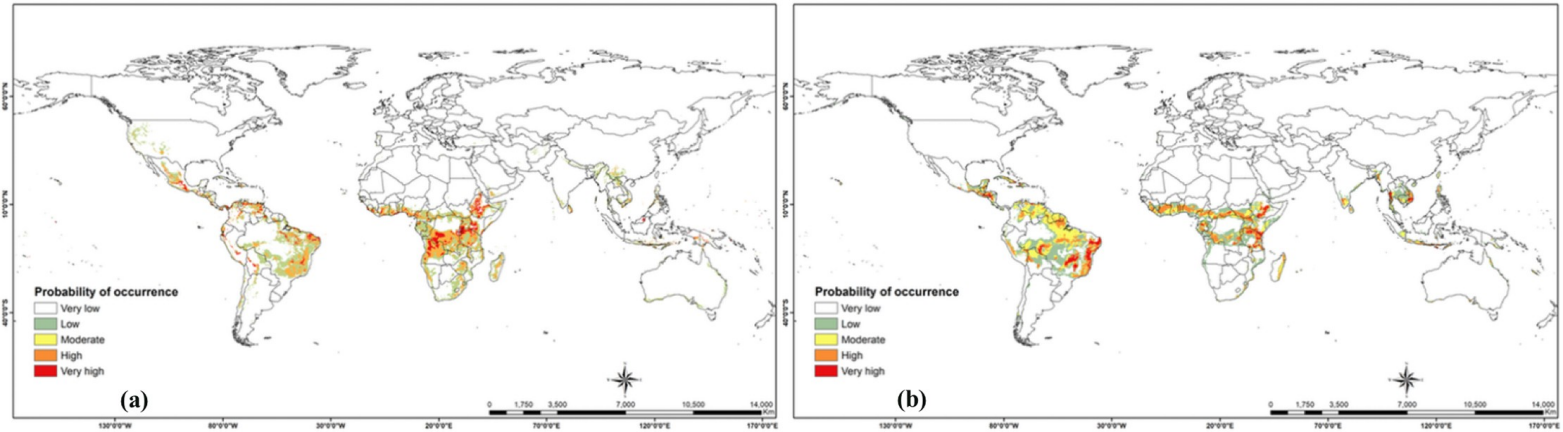
Future global habitat suitability of *Cotesia icipe*. Predicted future global potential distribution of *C*. *icipe* under climate change scenario: (a) MaxEnt model future global species distribution projections; (b) GARP model future global species distribution projections. “The figure was generated using the MaxEnt 3.4.1 software (https://biodiversityinformatics.amnh.org/open_source/maxent/) and GARP (http://openmodeller.sourceforge.net/)” [38].

The future projection for the *C*. *icipe* distribution in Africa indicates that the habitat suitability would remain similar to the current potential distribution or slightly contract towards the equator by the year 2050 with fewer novel suitable areas being identified (Fig 5A and 5B). Globally, overlaying the current and future *C*. *icipe* habitat suitability identified areas where *C*. *icipe* could potentially occur in the future (Fig 6A and 6B).

## Discussion

Alien invasive pests typically arrive in the new invaded region without their co-evolved efficient natural enemies, in what is referred to as the enemy release hypothesis [44–47] and fall armyworm, *S*. *frugiperda* a recent invader on the African continent is not an exception [7]. The logical and ideal approach is to search for an efficient natural enemy from the aboriginal home of the pest and reunite it with the pest in the new invaded country(ies) in what is commonly known as classical biological control. Indeed, several efficient parasitoid species have been recorded to attack this pest in its native home range [48–50]. However, classical biological programmes very often can be a lengthy process owing to bureaucracy on both sides of the exporting and importing countries [51]. Furthermore, most if not all importing countries require information regarding indigenous parasitoid diversity and their potential role on the invasive pest before considering introducing any exotic natural enemy. In this regard, following the detection and widespread occurrence of FAW in East Africa, and in an attempt to identify an indigenous parasitoid that could form a new association with this pest, we evaluated the performance of the newly described species, *C*. *icipe* against all the immature stages of *S*. *frugiperda*. Our findings show that *C*. *icipe* is unable to parasitise the egg and pupal stages. Also, the acceptance of *S*. *frugiperda* larval stage for oviposition varied with larval instar. In general, the parasitoid prefers the early instars (1^st^ and 2^nd^) for egg-laying. Three arguments can be put forward for this differential acceptability among the instars: (1) *C*. *icipe* females perceived that the older larvae (4^th^–6^th^ instar) to be of lower quality, in terms of host immunological defence against the development of its offspring; (2) the older larvae can physically defend themselves against the parasitoid; (3) the combination of both host quality and host defence. Evidence supporting the first argument in newly established host–parasitoid associations is abundant [52–56]. Physical defence by the larger size host has also been documented for several *Cotesia-*host system species [57, 58]. Similar findings of differential host stage acceptability have been reported for other related parasitoid species. For example, the acceptance by the congeneric *Cotesia glomerata* (L) (Hymenoptera: Braconidae) for its 6 host stages (early and late 1^st^, 2^nd^ and 3^rd^ instars) of its three Pierid hosts (Lepidoptera) was higher for the 1^st^ than for the 2^nd^ and 3^rd^ instars [59]. Likewise, in the same study, the authors demonstrated that *Cotesia rubecula* (Marshall) (Hymenoptera: Braconidae) females exhibited a significant preference for early 1^st^ instar of *P*. *brassicae* and *P*. *napi*. However, the same parasitoid failed to distinguish among six host stages of *P*. *rapae*. Kawaguchi and Tanaka documented a host-age preference for *Cotesia vestalis* (Hymenoptera: Braconidae), where parasitism decreased with host age when the females were offered different larval instars of the diamondback moth, *Plutella xylostella* (L.) (Lepidoptera: Plutellidae) [59].

The result of the suitability of various instars of FAW evaluated in this study for *C*. *icipe* development mirrored that of host acceptability; that is, the most accepted instars (1^st^ and 2^nd^) are the most profitable for the parasitoid in terms of the number of cocoons formed, as well as the number of emerged parasitoid wasps. Host acceptability, being an indicator for host suitability, is a well-documented phenomenon for *Cotesia* species [51, 52, 60], as well as other braconids [56, 61–63]. Our result of better performance of *C*. *icipe* on the earlier instars is in line with that reported for the related species, *C*. *vestalis*, whereby the per cent emerged wasps of this parasitoid were higher on 2^nd^ and 3^rd^ instars than on the 4^th^ instars of its host, *P*. *xylostella* [59].

The nature of the interaction of the members of the genus *Cotesia* and different host stages of their respective hosts in terms of developmental duration is intriguing. In this study, the developmental duration of *C*. *icipe* increased with host age with parasitism at 4^th^ larval instar resulting in longest developmental time, a finding that is in line with that reported for other congenic *Cotesia*. For example, *C*. *rubecula* developmental duration increased with the age of larval instar at parasitism of its host, *P*. *brassicae* [64]. Likewise, Harvey [65] reported that the developmental time of *C*. *glomerata* reared from different larval instars (1^st^, 2^nd^, and 3^rd^) of *P*. *rapae*, was longer when reared on 3^rd^ instar. However, in the same study, the researcher reported that the developmental duration of the same parasitoid decreased with the age of larval instar of initial parasitisation when reared on *P*. *brassicae*.

Similarly, *Cotesia ruficrus* (Haliday) parasitising the 4^th^ instar of its host *Cnaphalocrocis medinalis* (Guenée) (Lepidoptera: Pyralidae), had the shortest developmental duration than those parasitising at 2^nd^ and 3^rd^ [66]. Ngi-Song et al. and Jiang et al. also documented a shorter developmental time of parasitoids with initial parasitism at late instar in comparison to that of the early instar for C. *flavipes* [52, 67] and *C*. *vestalis* [58, 68]. A possible reason for the prolonged developmental duration in the later instars (as observed with *C*. *icipe* in this study, when reared on 4^th^ instar), has been attributed to the larger body mass of the developing wasp; it requires more time to consume it than that of the small-sized hosts (1^st^, 2^nd^ and 3rd in this case) [69]. Indeed, the parasitised 1^st^, 2^nd^ and 3^rd^ instars used in this study had their feeding reduced; and hence, their subsequent development. A large body of literature has documented that the same parasitoid species is able to parasitize different host species. In vast body of literature, it is clearly documented that the same parasitoid species parasitizing different host species and the same host parasitized by related *Cotesia* species may exhibit different developmental strategy [64, 65]. This calls for thorough for further study for better understanding of the bionomics of the members of this fascinating genus.

In arrhenotokous parasitic hymenopterans, sex allocation is known to be governed by host quality [70–72]. In the current study, the progeny of *C*. *icipe* was female-biased when reared on early larval instars. At the same time, it was male-biased when reared on late instars, in line with the findings by Nofemela [58] who reported that *C*. *vestalis* produced more female offspring when it parasitised 2^nd^ and 3^rd^ instars of *P*. *xylostella* compared to 4^th^ instar, on which the sex ratio was male-biased. However, Tanaka and Kawaguchi [73] reported comparable sex ratio for the same parasitoid on the same larval stage of *P*. *xylostella*.

Among other traits, Jervis and Ferns have recognised the ovigeny index (OI) as a key insect fitness parameter [73]. Moreover, Jervis et al. also considered ovigeny to play an important role in parasitoid–host population dynamics, and consequently, in pest management [41]. Our finding that *C*. *icipe* eclosed with a substantial number of mature eggs and continued to mature a few more eggs with age, established the pro-synovigenic nature of this parasitoid. The ovigenic index of 0.53 further confirms this, being the ratio of the initial egg load to the potential lifetime egg complements as defined by Jervis [41]. Although some of the members of the genus *Cotesia*, such as *C*. *flavipes* [74] and *Cotesia congregata* (Say) (Hymenoptera: Braconidae) [75, 76] are pro-ovigenic, Jervis et al. and Riddick reported similar pro-synovigeny to that for *C*. *icipe* in this study for *Cotesia marginiventris* (Cresson) [77], and *C*. *vestalis* [41]. With regards to the parasitoid fitness, Jervis and Ferns have argued that such reproductive strategy (OI < 1) is superior to absolute pro-ovigeny (OI = 1) [78]. The slight decline of the egg load of the nine-day-old wasps could be due to egg resorption, since the wasps were host-deprived. Researchers have argued that resorption of eggs by host-deprived females is considered an adaptive trait to preserve their energy [79] for other fitness traits, such as longevity [72, 80] possiby awaiting the availability of hosts.

In light of the results of this study, we can conclude that *C*. *icipe* is a pro-ovigenic, koinobiont, solitary, larval parasitoid. We can also deduce that the African indigenous parasitoid, *C*. *cotesia*, can form new associations with the alien pest, *S*. *frugiperda* and can be a promising candidate if used augmentatively within the context of a holistic IPM and contribute to the suppression of the FAW population.

However, it should be noted that this parasitoid was recovered from a vegetable ecosystem and a study on specificity needs to be undertaken to ascertain its comparative attractiveness to FAW infesting maize and other cereals, and other armyworm species infesting vegetables. Nevertheless, under the African setting (except North Africa) this might not be a serious hurdle, as maize fields are often small and intercropping vegetables with maize is a common practice. Moreover, recent studies revealed that *C*. *icipe* is among the common parasitoids recovered from FAW in the field [30, 31]. However, this does not preclude the significance of understanding the habitat specificity of *C*. *icipe*, as this parasitoid may have a differential attraction to different plants and host insects within the same arena. Indeed, Degen [81] reported differential parasitism of FAW by *Campoletis sonorensis* (Cameron) (Hymenoptera, Ichneumonidae, Campopleginae) and *C*. *marginiventris* on six different maize inbred lines.

Additionally, the parasitoid seems to be stage-specific, and release of other parasitoids that target older instar larvae (as well as egg and pupal stages) could complement the action of *C*. *icipe*. Further, *C*. *icipe* being new to science, this study lays the foundation of understanding the bionomics of this parasitoid, which aids its mass rearing. It also provides insights on the nature of interactions with the alien invasive pest, *S*. *frugiperda*, as well as the potential role of this parasitoid as a biocontrol agent for this pest.

In this study, we estimated the current and future potential distribution of *C*. *icipe* using MaxEnt and GARP algorithms. The habitat-suitability maps displayed slight differences between results obtained by the two approaches, under the present (Figs 3 and 4) and future climate scenarios (Figs 5 and 6). The result of habitat suitability of *C*. *icipe* evaluated in this study mirrored that of the host distribution [82]. More importantly, a highlight of suitable habitats will assist decision-makers in prioritising potential ‘hotspots’ for targeted release of the parasitoid. Our results suggest that under current climate scenarios, large areas of eastern and Central Africa are suitable for the parasitoid establishment. In West Africa, the suitability is restricted to the coastal regions of Nigeria, Togo, Ghana, Benin, Senegal, Ivory Coast and Sierra Leone. Additionally, countries such as South Africa, Madagascar, Namibia, Mozambique, Zambia, and Zimbabwe provide a suitable niche for the parasitoid establishment under current climatic conditions. Similar surveys confirmed the presence of *C*. *icipe* in Ghana, Benin, Senegal and Zambia, thereby validating the accuracy of our model [83, 84]. At the same time, there is a moderate likelihood of establishment and colonisation in North Africa, due to abiotic factors, such as physical barriers (Sahara Desert) that might impede dispersal of the parasitoid. Early et al. [81] have also predicted that most of the areas identified to be suitable for *C*. *icipe* are suitable for FAW establishment and development in eastern Africa, highlighting its potential for conservation biological control. However, in West Africa, the suitability for FAW establishment lies much beyond the coastal regions [82]. Hence, the need to identify more suitable parasitoids in West and North Africa exists. Overall, the Maxent model (AUC = 0.997 ± 0.001) predicted broader potential area for the parasitoid establishment compared to the GARP model and was consistent with the recent surveys conducted in Ghana, Benin and Zambia [83, 84].

While the future potential distribution of *C*. *icipe* is similar to the current potential distribution, we observed a slight contraction towards the equator due to the potential effects of global climate change, such as increased temperatures, under future climate scenarios. This seems to be in line with future projection of the FAW and host plant (maize) [85, 86]. The parasitoid model suggests that the habitat suitability would minimally expand under predicted levels of climate change. Therefore, the parasitoid does not appear to undergo a significant niche shift under climate change scenario and offers the most plausible option for the biological control and management of the FAW. A highlight of suitable habitats for the parasitoids in this study will assist in prioritising potential parasitoid release locations.

## Conclusions

In the light of our results, it is clear that the African indigenous parasitoid, *C*. *icipe* has formed a new association with the alien invasive pest, *S*. *frugiperda*. The potential distribution and establishment of this parasitoid in most parts of Africa are indications of the possible success in using the parasitoid in both augmentative and conservation biocontrol of FAW in Africa. Moreover, the high reproductive rate and short generation time of *C*. *icipe* [29] coupled with ease of mass production, make *C*. *icipe* an ideal candidate for augmentative releases, an activity which we are currently undertaking. Indeed, other related micrograstrinae parasitoids have been used successfully in augmentative and conservation biocontrol of other lepidopteran pests in Africa. For example, *C*. *flavipes*, as a bicontrol agent against the invasive pest, *C*. *partellus* in Kenya, Uganda and Tanzania [19, 87, 88]. Similarly, in New Zealand, the success of augmentative release of exotic *Cotesia urabae* Austin and Allen (Hymenoptera: Braconidae) to control the invasive *Uraba lugens* Walker (Lepidoptera: Nolidae) shows the potential of microgastrinae in managing invasive pests beyond the parasitoids’ aboriginal homes [20]. Notably, the success of using these parasitoids as biocontrol agents against various pests is dependent on the conservation strategies for natural enemies, which are put in place by the farmers, such as reduced application of pesticides and intercropping. In addition, the Maxent model (AUC = 0.997 ± 0.001) predicted broader potential areas for the parasitoid establishment in tropical and subtropical regions of Africa compared to the GARP model and this observation was consistent with the recent surveys conducted in Ghana, Benin, and Zambia. More importantly, the models highlighted potential ‘hotspots’ for targeted release of the parasitoid.

## Supporting information

S1 File Host stage acceptability data for *Cotesia icipe*. (XLSX)

S2 File *Cotesia icipe* percent cocoon formed per host stage. (XLSX)

S3 File *Cotesia icipe* percent female progeny per host larval stage. (XLSX)

S4 File Number of *Cotesia icipe* emerged wasps per host larval stage. (XLSX)

S5 File Time to cocoon formation of *Cotesia icipe* in different host larval stages. (XLSX)

S6 File *Cotesia icipe* Developmental time host larval stage. (XLSX)

S7 File Egg load and ovigeny index of *Cotesia icipe*. (XLSX)
